# Dietary supplementation with a blend composed of carvacrol, tannic acid derived from *Castanea sativa* and *Glycyrrhiza glabra*, and glycerides of medium chain fatty acids for weanling piglets raised in commercial farm

**DOI:** 10.1007/s11259-024-10539-1

**Published:** 2024-09-13

**Authors:** Luca Marchetti, Raffaella Rebucci, Davide Lanzoni, Carlotta Giromini, Lucia Aidos, Alessia Di Giancamillo, Paola Cremonesi, Filippo Biscarini, Bianca Castiglioni, Valentino Bontempo

**Affiliations:** 1 Department of Veterinary Medicine and Animal Sciences, Università degli Studi di Milano, Lodi, 26900 Italy; 2 Department of Biomedical Sciences for Health, Università degli Studi di Milano, Milano, 20100 Italy; 3 Institute of Biology and Biotechnology in Agriculture, National Research Council (CNR), Lodi, 26900 Italy

**Keywords:** Feed additive, Microbiota, Gut health, Weaning

## Abstract

This study aimed to evaluate the dietary administration of a blend composed of carvacrol, tannic acid derived from *Castanea sativa* mill and *Glycyrrhiza glabra*, medium chain fatty acids (MCFAs) glycerides for weanling piglets. An in vitro digestion followed by total phenolic content (TPC) and total antioxidant activity (TAC) assessment was performed before the in vivo application. At weaning, a total of 210 piglets were randomly allocated to two experimental treatments (7 replicates/15 piglets for each replicate). Control group (CTR) was fed a standard basal diet while the treated group (T) was fed the basal diet mixed with 1.500 mg/kg of blend. After in vitro digestion, TPC and TAC evidenced peaks at the end of oral and gastric phases in comparison to the intestinal one in line with the high content of phenolic compound (*P* < 0.05). Treatment conditioned body weight and average daily gain (*P* < 0.05), fecal score on 6, 7, and 8 d after weaning (*P* < 0.05). At 35d, the T group showed a decrease in salivary cortisol compared to CTR (*P* < 0.05). Duodenum and jejunum sections of T piglets revealed higher villi (*P* < 0.05), deeper crypts (*P* < 0.01), and increased V/C ratio (*P* < 0.01). CTR showed a higher expression of duodenal Occludin (*P* < 0.05). Jejunal E-cadherin and Occludin were more expressed in T jejunum sections (*P* < 0.05). Twelve differentially abundant genera were identified in T group caecal samples. Potentially harmful *Clostridium sensu stricto 13* was reduced by the treatment (*P* < 0.05). In conclusion, the tested blend positively affected salivary stress markers and the gut health of weaned piglets.

## Introduction

Weaning is a process accompanied by notable changes in intestinal morphology, especially regarding villus height and crypt depth, caused by transient anorexia (Lallès et al. 2007). Moreover, the impairment in terms of digestive enzymes production at this stage contributes in promoting the accumulation of unabsorbed nutrients in the large intestine where potential pathogenic bacteria such as enterotoxigenic strains of *Escherichia coli* that could further favor the onset of post-weaning diarrhea (PWD) (Fairbrother et al. 2005). Furthermore, this threat is exacerbated by the lack of an adequate immune status in weanling piglets, which potentially undergo chronic and acute inflammatory status (Pié et al. 2004). Consequently, PWD represents the most harmful condition during the weaning transition of piglets promoting a detrimental situation in economic terms, characterized by high morbidity and mortality, veterinary interventions, labour costs and negative reflexes on productive parameters (Laird et al. 2021). After the European Community ban on antimicrobials as growth promoters in 2006, pharmacological dosages (2000–3000 mg/kg of complete feed) of Zinc Oxide (ZnO) represented a widely diffuse strategy to promote gut health in weanling piglets by avoiding PWD (Corino et al. 2021). However, starting from June 2022 the European Commission decided to ban the prescription of ZnO oral medication for livestock (Bonetti et al. 2021). The concerns that led to the ban of high dosages of ZnO in feed were linked to the low bioavailability of this trace element and, consequently, to its environmental impact, but also to the co-selection of antibiotic-resistance bacterial strains (Mantovi et al. 2003). Therefore, the research of valid alternatives able to enhance gut health without having negative effects in terms of pollution or safety is strictly needed.

Active compounds from natural extracts could block the activation of both inflammation and oxidative stress signal pathways (Galli et al. 2020; Na and Surh 2008). In this sense, one of the most studied compounds is carvacrol which has a large spectrum of antimicrobial activities against gram-negative and gram-positive bacteria (Roller and Seedhar 2002). Briefly, this antimicrobial activity is determined by the presence of the hydroxyl group in the molecule of the natural compound, which contributes to the release of bacterial lipopolysaccharides (LPS) from gram-negative membrane. Furthermore, among polyphenolic compounds, tannic acid (TA) proved to be useful in positively modulating the intestinal microbiota, improving energy metabolism through higher production of volatile fatty acids (VFA), and increasing the integrity of the intestinal barrier (Song et al. 2021). Moreover, the administration of medium and short-chain fatty acids (MCFAs) could also represent a valid tool for enhancing gut health during the weaning transition (Chen et al. 2019). Indeed, it is recognized how molecules like lauric acid (C:12), capric acid (C:10), and caprylic acid (C:8) could inactivate pathogens proliferation both by promoting the acidification of the intestinal environment or acting against the expression of virulence factors. In addition, the inclusion of low dietary levels of MCFAs showed modulatory effects on the enteric microbiota population (Omonijo et al. 2018) and demonstrated positive reflexes in terms of gut morphology on villus height, and tight junctions proteins (TJs) in the proximal tract of the small intestine (Zentek et al. 2011). Moreover, the administration of glycerides of fatty acids has been reported to control pathogens proliferation and reduce post-weaning diarrhea (Correa et al. 2021). Therefore, despite widely diffused knowledge on the effects of tannic acid, MCFAs and carvacrol (Song et al. 2021; Lauridsen 2020), little is known about the possible effects on the gut health of piglets when fed a blend obtained by these single active compounds. The critical aspect of blends is probably linked to the possible interaction between the different components that may affect their efficacy in conditioning the gut environment (Canibe et al. 2022). However, different composition blends exerted positive reflexes on the gut health of weanling piglets raised in experimental facilities (Rebucci et al. 2022; Luise et al. 2023). Nevertheless, testing these blends in commercial farm conditions could reveal different effects and new insights. Indeed, the exposure to typical stressors of the weaning transition combined with the higher density of commercial farms could make piglets more easily vulnerable to PWD. For these reasons, the study aim was to investigate the effects of the dietary administration of a blend composed of carvacrol, TA and MCFAs on the gut health of weanling piglets raised in commercial farm conditions.

## Materials and methods

### In vitro digestion, total phenolic content and antioxidant capacity

In vitro digestion was performed as reported by Regmi et al. (2009) with minor modifications introduced by Lanzoni et al. (2023). At the end of each digestion step, aliquots (about 1 mL) were taken and frozen immediately at -20 °C and used to measure total phenolic content (TPC) and antioxidant activity. In parallel, at the end of digestion, the samples were filtered using paper filters (Whatman 54 Florham Park, NJ), thus obtaining the undigested fraction (UF). Subsequently, the filters were dried overnight at 65 °C. Then, the dry matter digestibility (% dry matter; DM) was measured using the following formula:

$$\:DM\:Digestibility=\left[\frac{DMx-DMy}{DMx}\right]*100$$

Where DMx is the sample dry matter percentage, while DMy represents UF dry matter percentage.

For TPC, the protocol of Attard (2013) was used with minor adaptations as reported by Lanzoni et al. (2023). Tannic acid, methanol, Folin–Ciocalteu (FC) reagent, and sodium carbonate were purchased from Sigma Chemical Co. (St. Louis, MO, USA). Tannic acid was prepared in a 1:2 diluition. FC reagent was diluted with distilled water (1:10); contextually 1 M solution of sodium carbonate was prepared. Then, 100 µL of each sample (7 aliquots) was added to 500 µL of FC and 400 µL of sodium carbonate and incubated at room temperature for 20 min. At the end of the incubation period, samples were read at 630 nm. TPC Values were expressed in terms of tannic acid equivalent (mg TAE/100 g of dried samples). The FRAP assay was performed following the protocol of Abdelaleem and Elbassiony (2020), with minor modifications. FRAP values are expressed as mg FeSO_4_/100 g of dried sample. The antioxidant activity was assessed using the ABTS method according to the protocol of Re et al. (1999) with minor adaptation. For TPC, FRAP and ABTS oral, gastric and intestinal phase were considered. Values were expressed in terms of Trolox equivalent (mg TE/100 g of dried samples). Analyses were performed on biological and technical triplicate for each parameter.

### Animals housing and experimental design

The in vivo trial was performed at Azienda Agricola Pianoverde of Santorelli-Brontesi S.S., Boarini Farm, Via Cascina, 25,023 Leno (Brescia). At weaning, corresponding to 0 d of the trial, 210 cross-bred twenty-eight-day old piglets (Stambo HBI X Dalland 40) were randomly distributed, according to their body weight, into the treatment and control experimental groups (105 each). Each group was replicated seven times, with 15 piglets per pen forming the experimental unit. Animals were housed in two different rooms of 7 replicates each and 1 pen at disposal as infirmary. Trial lasted 35 days corresponding to 61 d old piglets. Each pen had a slatted floor and was fitted with a stainless-steel feeder and nipple waterers. The rooms were lit by a combination of daylight and artificial light. Rooms temperature, humidity, and air quality were automatically controlled. Ventilation was achieved by two, variable-speed fans linked to temperature sensors. The temperature inside the building was approximately 28 °C at the start of the trial and was adjusted weekly until a final temperature of 24–25 °C was achieved. The relative humidity was settled between 60 and 70%. Piglets had water and feed available *ad libitum*.

The control group (CTR) was fed a basal diet, whereas the treated group (T) was fed the basal diet mixed with a dosage of the product corresponding to 1.500 mg/kg. For the treated diet, the proper quantity of additive was weighed using a balance and premixed with a small amount of the feed as a carrier, before adding this to the final mix to ensure homogeneous distribution in the complete feed. All diets were formulated to meet or exceed the nutrient requirements recommended by the NRC (2012) for post-weaning piglets (Table 1). The treatment compound was a blend of 5% of carvacrol, 23% of monoglycerides, diglycerides and triglycerides of medium chain fatty acids (capric, caprylic and lauric acid) and tannic acid derived from 26% of *Castanea sativa* mill and 2% of G*lycyrrhiza glabra* extract stabilized on silica (Gastroherb Plus produced by Phytsolutions, Caldes de Montbui, Barcelona, Spain).

**Table 1:** Composition and chemical analysis of the post-weaning diets (% on dry matter basis). DM: dry matter; CP: crude protein; EE: ether extract; CF: crude fibre; ca: calcium; P: phosphorus; NE: net energy

Ingredients, % as fed	Pre starter (0–14 d)	Starter (14–35 d)
Barley	19.50	18.50
Corn	24.00	24.00
Wheat	9.00	10.00
Dry whey	5.00	4.00
Soybean oil	2.00	2.00
Fish meal	2.00	-
Bakery by-products (10% CF)	10.00	8.00
Soybean meal 48% CP	6.50	8.50
Extruded soybean	4.00	6.00
Toasted soybean	3.75	3.75
Vitamin and mineral premix^a^	0.25	0.25
Flacked barley	3.00	3.00
Soybean concentrated	2.00	2.00
Soybean oil	1.00	1.00
Calcium carbonate	1.20	1.20
Monocalcium phosphate	0.60	0.60
Sodium chloride	0.20	0.20
Bran	4.00	5.00
Beet pulp	2.00	2.00
Chemical analysis, % DM	Pre starter (0–14 d)	Starter (14–35 d)
DM, %	88.00	88.00
CP, %	17.46	17.65
EE, %	6.57	6.61
CF, %	3.23	3.42
Ash, %	5.72	5.55
Ca, %	0.89	0.83
P, %	0.55	0.53
NE, kcal/kg	2.471	2.469
Lysine, %	1.20	1.20
Methionine, %	0.43	0.42
Methionine + Cysteine, %	0.72	0.72
Threonine, %	0.78	0.78
Tryptophane, %	0.23	0.23

^a^Additives ( per Kg ): Vitamin.pro-vitamin and analogue substances:3a672a Vitamin A 63.193 UI3a671 Vitamin D3 13.226 UI3a700 Vitamin E (All-rac-alfa-tocoferile acetate) 353 mg3a711 Vitamin K3 14.7 mg3a821 Vitamin B1 14.7 mg3a825ii Vitamin B2 47.0 mg3a831 Vitamin B6 14.7 mg Vitamin B12 0.29 mg3a314 Niacin 294 mg3a841 Calcium D-pantothenate 245 mg3a316 Folic Acid. 5.1 mg3a880 Biotin 0.59 mg3a890 Choline Chloride 903 mg Trace elements: E4 Copper (3b412 Copper oxide [I].) 476 mg E4 Copper (3b405 Copper sulphate[II] pentahydrate.) 113 mg E1 Iron (3b103 Iron sulphate [II] monohydrate.) 1.344 mg E 2 Iodine (3b202 Calcium iodate anhydrous.) 11.2 mg E5 Manganese (3b502 Manganese oxide [II].) 560 mg E 8 Selenium (3b801 Sodium selenite.) 2.8 mg E6 Zinc (3b603 Zinc oxide.) 504 mg Preservatives: E330 Citric acid 1.1 mg1a297 Fumaric acid 5.300 mg Antioxidant: E310 Propyl gallate 0.37 mg E321 Butylhydroxytoluene (BHT) 118 mg Binders: E551a Silicic acid 2.5 mg E563 Sepiolite 5.491 mg Digestion promoters:4a16 6-phytate (EC 3.1.3.26) 2.118 OTU4a1617 Endo-1.4-beta-xilanase EC 3.2.1.8 19.980 EP

### Growth parameters, fecal score and general health

The body weight and feed consumption were measured at 0, 14, and 35 days. Feed was distributed daily in trails after being weighed by a scale and having registered the weighted quantity. Therefore, average daily feed intake (ADFI) and average daily gain (ADG) were calculated for the 3 different periods of the trial (0–14 d, 14–35 d, and 0–35 d). Feed conversion rate (FCR) and feed efficiency (FE) were subsequently obtained by ADFI/ADG and ADG/ADFI ratios respectively. Body weight (BW) and ADG were also registered accounting for single animals. Mortality, pathologies, or unusual adverse events were recorded daily. Fecal score evaluation was performed daily from 0 d to 35 d on trial through a 0 to 4 scale (0 = normal stool, 4 = diarrhea) as reported by Ruckman et al. (2020).

### Salivary cortisol level, immunoglobulins a (IgAs), and total antioxidant capacity (TAC)

Saliva samples were taken on days 14, 21, and 35 of the trial using Salivette^®^ tubes (Sarstedt AG& Co., Germany) from one subject per replicate. A cotton swab was kept in the mouth of the animal for 1–2 min following the procedures described by Escribano et al. (2019). The cotton swab was then placed in the tube and centrifuged at 3000 rpm for 13 min. The saliva aliquots obtained by centrifugation were then stored in Eppendorf tubes (Sarstedt AG& Co., Germany) and frozen at − 80° C until analysis.

Cortisol and IgAs were quantified using competitive and sandwich ELISA kit tests according to the manufacturer’s instructions (Immunological sciences, Società Italiana Chimici, Rome, IT). IgAs and Cortisol values are expressed in ng/ml. The total antioxidant capacity (TAC) evaluation was performed through ferric reducing antioxidant power (FRAP) method performed with a commercial kit (Elabscience Biotechnology Co.,Ltd). Values of FRAP are reported as µmol/l of Trolox equivalents.

### Intestine histology and histometry

At the end of the trial, animals were slaughtered, and intestinal tissue sampling was performed (*n* = 7 per group). Portions of 1 cm^3^ of the small intestine (duodenum 2 cm after the pylorus and duodenojejunal junction, according to Ishida et al. 2018) were immediately collected and fixed in 10% neutral buffered formalin for 24 h at 4 °C, dehydrated in a graded series of ethanol, cleared with xylene, and embedded in paraffin. Microtome Sect. (4 μm thick) of both duodenum and jejunum were stained with Hematoxylin–Eosin (HE) to establish structural details. On these HE-stained sections, the height of intestinal villi (V) (10 villi measured per section) and the depth of intestinal crypts (C) (10 crypts measured per section) were measured and calculated by image analysis software (Proview, Optika, Italy). The ratio between villi and crypts (V/C) was also calculated.

### Gut barrier assessment: E-Cadherin, Zonulin-1, and Occludin immunofluorescence staining

Other sections of the duodenum and jejunum were used for immunofluorescence. Briefly, after rehydration, heat-induced antigen retrieval was performed (citrate buffer pH 6, 5 min microwaves 600 W, followed by cooling, twice). After washing three times in Phosphate buffer saline (PBS, pH 7.4), treatment with the Avidin–Biotin blocking kit solution (Vector Laboratories Inc., Burlingame, CA USA) was performed. Sections were incubated with the primary antiserum: anti-E-Cadherin (E-CAD; 1:30, ab15148, Abcam, UK), zonulin-1 antibody (ZO-1, 1:100, Cat. No. GTX108592, GeneTex, USA), and anti-Occludin antibody (1:100, ab216327, Abcam, UK), for 24 h at room temperature and washed in PBS. Afterward, sections were incubated with 10 µg/ml goat biotinylated anti-rabbit IgG (Vector Laboratories Inc., Newark, USA) for 2 h at room temperature. After rinsing twice in PBS, the sections were treated with Fluorescein–Avidin D (Vector Laboratories Inc., Newark, USA), 10 µg/ml in NaHCO3, 0.1 M, pH 8.5, 0.15 M NaCl for 2 h at room temperature. Finally, slides with tissue sections were embedded in Vectashield Mounting Medium with DAPI (SKU H-1200-10, Vector Laboratories Inc., Newark, USA) and observed using a Confocal Laser Scanning Microscope (FluoView FV300; Olympus). The immunoreactive structures were excited using Argon/ Helio–Neon–Green lasers with excitation and barrier filters set for rhodamine. Images containing superimposition of fluorescence were obtained by sequentially acquiring the image slice of each laser excitation or channel. The absence of cross-reactivity with the secondary antibody was verified by omitting the primary antibody during the first incubation step.

For the quantification of each of the three immunofluorescence, duodenum, and jejunum sections were examined using the FluoView software for image analysis (Olympus). Excitation, and barrier filters were set for rhodamin. The laser power and photomultiplier tube voltage were constant so that the fluorescence intensities of various samples could be compared. Images were digitized under constant gain and laser offset, with no post-capture modifications. Five section areas of the epithelium that contained the largest and brightest immunofluorescence for each sample were selected for measurement. The areas to be assessed were defined manually and used to normalize each peak intensity. The calculated mean fluorescence intensity was obtained for each of the selected section areas according to (Di Giancamillo et al. 2009). Pixel intensity was determined using the histogram/area functions of the FluoView software, which assigned the gray levels (GL) within a 0–256 Gy scale. Data were presented as mean fluorescence intensity.

### Caecal microbiota evaluation: sample collection and DNA extraction

At the slaughterhouse, caecal content samples were collected in sterile vials from 14 piglets (7 from CTR and 7 from T groups) and stored at − 80 °C until DNA extraction. DNA was extracted from each sample using the QIAmp Fecal Pro kit (Qiagen, Hilden, Germany), according to the manufacturer’s protocol. DNA quality and quantity were assessed using a NanoDrop ND-1000 spectrophotometer (NanoDrop Technologies, Wilmington, DE, USA), and then it was stored at − 20 °C until use.

### 16 S ribosomal RNA (rRNA) gene sequencing and bioinformatics processing

Bacterial DNA was amplified using the primers described by Caporaso et al. (2011) which target the V3-V4 hypervariable regions of the 16 S rRNA gene. All PCR amplifications were performed in 25 µL volumes per sample. A total of 12.5 µL of KAPA HIFI Master Mix 2× (Kapa 344 Biosystems, Inc., MA, USA) and 0.2 µL of each primer (100 μm) were added to 2 µL of genomic DNA (5 ng/µL). Blank controls (no DNA template added to the reaction) were also performed. A first amplification step was performed in an Applied Biosystem 2700 thermal cycler (ThermoFisher Scientific). Samples were denatured at 95 °C for 3 min, followed by 25 cycles with a denaturing step at 98 ◦C for 30 s, annealing at 56 °C for 1 min and extension at 72 °C for 1 min, with a final extension at 72 °C for 7 min. Amplicons were cleaned with Agencourt AMPure XP (Beckman, Coulter Brea, CA, 351 USA) and libraries were prepared following the 16 S Metagenomic Sequencing Library Preparation Protocol (Illumina, San Diego, CA, USA). The libraries obtained were quantified by Real Time PCR with KAPA Library Quantification Kits (Kapa Biosystems, Inc., MA, USA), pooled in equimolar proportion and sequenced in one MiSeq (Illumina) run with 2 × 250-base paired-end reads.

The 16 S rRNA gene sequences determined in this study were deposited in the NCBI Sequence Read Archive (SRA) database.

Demultiplexed paired-end reads from 16 S rRNA-gene sequencing were first checked for quality using FastQC (Andrews 2010). Reads were then cleaned by removing primers and adapters with the python tool Cutadapt (Martin 2011), and by trimming for quality using the C + + tool Sickle (Joshi and Fass 2011), with Phred threshold > 20 (i.e. the end part of the reads was removed if its quality deteriorated). After cleaning, forward and reverse paired-end reads were joined together using the python pipeline Micca (Microbial Community Analysis) (Albanese et al. 2015), specifically the function ‘mergepairs’ with default values (i.e. minimum overlap length = 32, maximum number of mismatches in the overlap region = 8). Assembled reads were filtered for quality, discarding reads with missing/uncalled bases or with an expected error rate larger than 1% (1 error in 100 bases). All remaining reads were used to identify OTUs (Operational Taxonomic Units) with the denoising approach (Rosen et al. 2012) implemented in the Micca function ‘out’ (method ‘denovo_unoise’). Finally, the identified OTUs were classified using the MICCA function ‘classify’ to assign taxa as annotated in the SILVA132 reference database (Glöckner et al. 2017) with the following parameters: maximum number of hits -taxa- to consider for each OTU = 3; assign taxon if present in at least 0.5 of the hits; reject OTU if the fraction of alignment to the reference sequence is lower than 0.75).

### Statistical evaluation

A One-way ANOVA was applied to analyze TPC, FRAP, and ABTS through in vitro digestion phases. All experimental data relative to growth performance, fecal score, salivary IgAs, TAC, and cortisol were analysed as a completely randomized block design by ANOVA using the MIXED procedure of SAS v. 9.2 (SAS Institute Inc., Cary, NC, USA) accounting for the effect of treatment, time, and their interaction. The model included the treatment, time, their interaction and the room. A general linearized model (GLM) was considered to evaluate duodenal and jejunal histometry and tight junction expression. When the data regarding tight junction expression were normally distributed, a One-Way ANOVA was performed, otherwise, the Kruskal-Wallis test was applied. These data were analyzed with GraphPad Prism 9.0.0 and are presented as means ± S.E.M. Pen represented the experimental unit for overall growth performances and fecal score. Individual piglet was considered as the experimental unit for single animal mean BW and ADG, as in the case of intestinal histometry and tight junction. Post-hoc evaluation was performed with a Bonferroni test. The OTU table obtained from 16 S rRNA-gene sequencing was first filtered to remove OTUs with less than 10 total counts distributed in fewer than 3 samples. Filtered OTU counts were then normalised for uneven sequencing depth by cumulative sum scaling (CSS; Paulson et al. 2013). The normalised OTU table was used to calculate the alpha (ACE, Chao1, Fisher’s alpha, Shannon, Simpson, Inverse Simpson) and beta (Bray-Curtis distances) diversity in the piglets gut samples. Details on the calculation of the alpha- and beta-diversity indices can be found in Biscarini et al. 2018 (Appendix S2). Differences between experimental groups (treatment and control) for OTU counts and alpha diversity indices were evaluated using analysis of variance. Differences between distance matrices were evaluated non-parametrically using the permutational analysis of variance approach (PERMANOVA with 999 permutations) (Anderson 2001). Results were considered statistically significant for *P* < 0.05 and highly significant for *P* < 0.01. Graphs were realized with GraphPad Prism 9.0.0.

## Results

### Health status and adverse events

During the first two weeks of the study, 6 CTR group piglets and 1 T group piglet were treated with 1 ml Enrofloxacin (100 mg/ml) as they had diarrheal disorders. In addition, seven piglets were excluded from the test for the presence of abdominal hernias (3 CTR and 4 T). All the treated / excluded piglets were moved to infirmary pens.

### In vitro digestibility: TPC, FRAP and ABTS assay

**Fig. 1:**
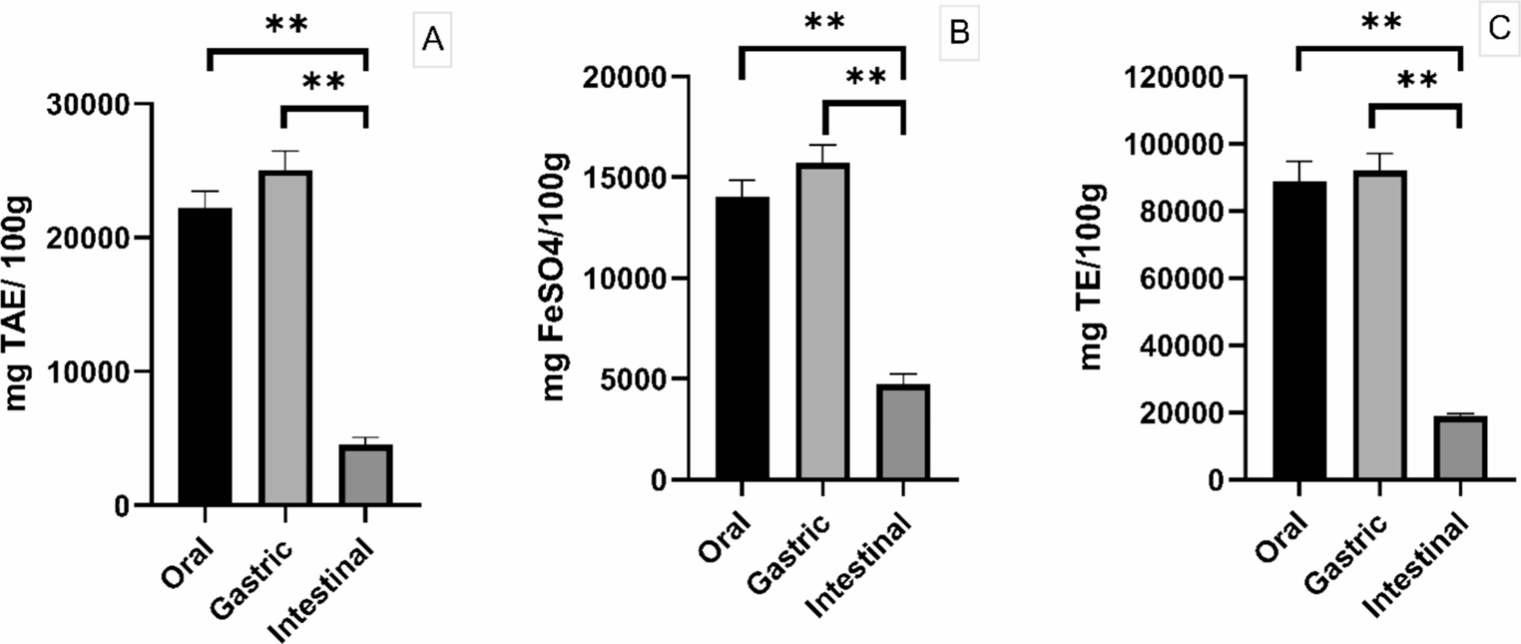
Total phenolic content (TPC), FRAP and ABTS results registered at the end of each digestion phase (A, B and C respectively). Values are expressed as mean ± standard error mean (S.E.M). * = *P* < 0.05, ** = *P* < 0.01

DM digestibility revealed a value of 49.68 ± 2.12%. Moreover, for TPC, FRAP and ABTS followed a similar trend through in vitro digestion process. Briefly, in oral, and gastric phase TPC values were higher than intestinal phase (22193.68 ± 1301.74 mg TAE/100 g and 25032.63 ± 1419.93 mg TAE/100 g vs. 4534.74 ± 549.13 mg TAE/100 g; *P* < 0.01, Fig. 1A). Similarly, oral, and gastric FRAP revealed the same trend in comparison with the intestinal phase (14048.32 ± 268.62 mg FeSO_4_/100 g and 15718.55 ± 297.06 mg FeSO_4_/100 g vs. 4727.07 ± 167.91 mg FeSO_4_/100 g; *P* < 0.01, Fig. 1B). ABTS values in oral and gastric phase were equally higher than intestinal one (88824.92 ± 6032.04 mg TE/100 g and 92081.07 ± 5104.48 mg TE/100 g vs. 18820.56 ± 894.27 mg TE/100 g; *P* < 0.01, Fig. 1C).

### Fecal score

**Fig. 2:**
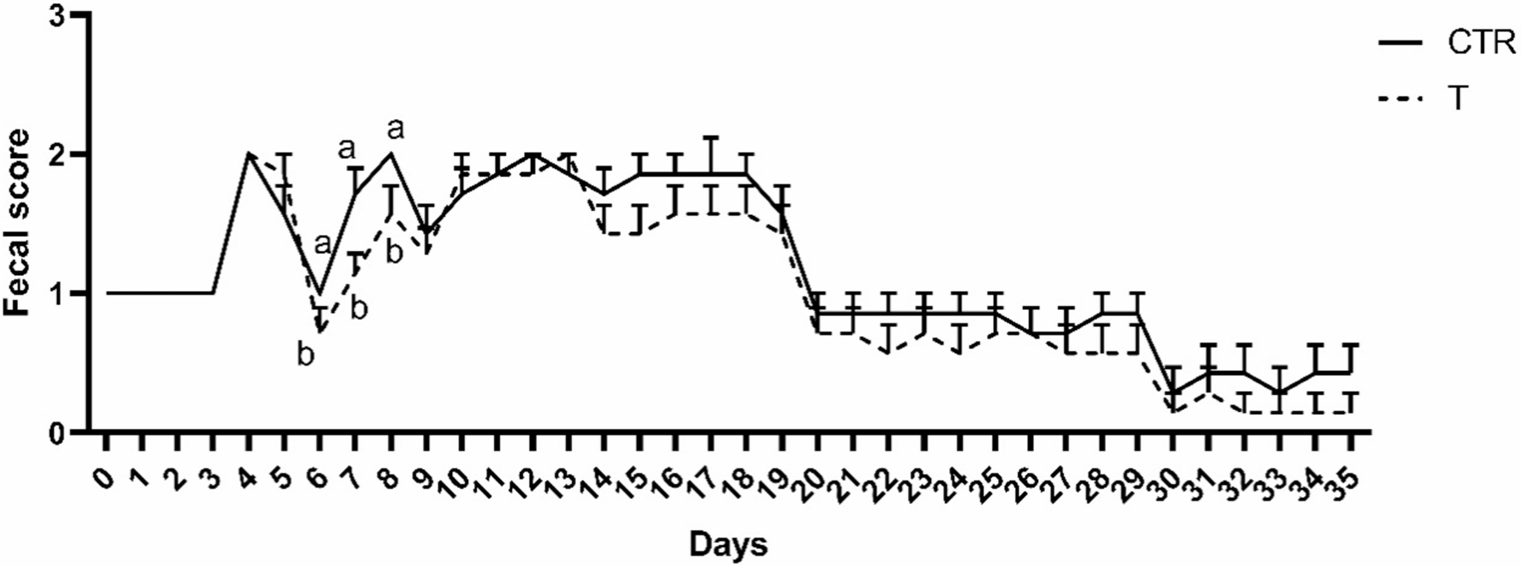
Fecal score evaluation performed during the trial (0–35 d) suggested a better consistency of feces of the T group compared to the CTR one from day 6 to 8 on trial. Values are presented as means ± standard error mean (S.E.M); different letters mean statistically significant results (a, b *P* < 0.05). CTR: control group; T: treated group

On days 6, 7 and 8 on trial, the treated group registered a better fecal consistency as depicted by the interaction between time and treatment variables (*P* < 0.05, Fig. 2). Furthermore, the administration of the tested blend was useful in ameliorating fecal score of treated animals even when considering the treatment as single variable (*P* < 0.05). Specifically, a difference between CTR and T was detected already at 6 d after weaning (1.00 ± 0.36 vs. 0.71 ± 0.48, *P* < 0.05, Fig. 2). Moreover, at 7 d CTR vs. T comparison revealed a better fecal score for the treated group (1.71 ± 0.38 vs. 1.14 ± 0.47, *P* < 0.05, Fig. 2). Finally, this difference was also underlined at 8 d on trial comparing CTR vs. T group (2.00 ± 0.46 vs. 1.57 ± 0.37, *P* < 0.05, Fig. 2).

### Growth performances

The growth performances of individual piglets are shown in Table 2 (individual weight and ADG), while the results related to pens as experimental units are shown in Table 3. Despite the lack of significance in the interaction between treatment and time, the treated group fed with carvacrol, TA, and MCFAs registered better growth performances during the trial. BW of T piglets was found to be higher compared to CTR (16.67 ± 3.13 kg vs. 15.82 ± 2.79, *P* = 0.041, Table 2). Average daily gain (ADG) also improved in the T group (0.25 ± 0.087 kg vs. 0.23 ± 0.078, *P* = 0.003, Table 2). On the other hand, data related to pens revealed a tendency towards better FCR and FE for the T group if compared to CTR (Table 3).

**Table 2:** Growth performance of single post-weaning piglets after the administration of carvacrol, TA, and MCFAs blend. All the values are intended as means ± standard error mean (S.E.M); BW = body weight; ADG = average daily gain. CTR: control group; T: treated group. Treatment, time and their interaction *p*-values are displayed in the *p*-value section of the table

Treatments			*p*-value			
Parameters	CTR	T	S.E.M	Treatment	Time	Time* Treatment
BW (kg)						
0 d	7.65	7.74	0.20	0.041	< 0.001	0.144
14 d	9.03	9.23				
35 d	15.82	16.67				
ADG (kg/d)						
0 d	0.10	0.11	0.01	0.03	< 0.001	0.259
14 d	0.32	0.35				
35 d	0.23	0.25				

**Table 3:** Growth performance of pens evaluated from day 0 to 35 on trial. All the values are intended as means ± standard error mean (S.E.M); BW = body weight; ADG = average daily gain; ADFI = average daily feed intake; FCR = feed conversion rate; FE = feed efficiency. CTR: control group; T: treated group. Treatment, time and their interaction *p*-values are displayed in the *p*-value section of the table

Treatments			*p*-value			
Parameters	CTR	T	S.E.M	Treatment	Time	Time*Treatment
BW (kg)						
0 d	111.52	111.62	9.81	0.574	< 0.001	0.797
14 d	131.64	133.17				
35 d	228.28	240.54				
ADG (kg/d)						
0–14 d	1.44	1.54	0.23	0.111	< 0.001	0.670
14–35 d	4.60	5.11				
0–35 d	3.34	3.68				
ADFI (kg/d)						
0–14 d	2.71	2.81	0.19	0.351	< 0.001	0.965
14–35 d	7.67	7.88				
0–35 d	5.69	5.85				
FCR						
0–14 d	1.95	1.86	0.08	0.091	0.005	0.953
14–35 d	0.60	0.65				
0–35 d	0.58	0.63				
FE						
0–14 d	0.53	0.54	0.02	0.087	0.003	0.828
14–35 d	0.60	0.65				
0–35 d	0.58	0.63				

### Salivary cortisol, IgAs, and total antioxidant capacity (TAC)

**Fig. 3:**
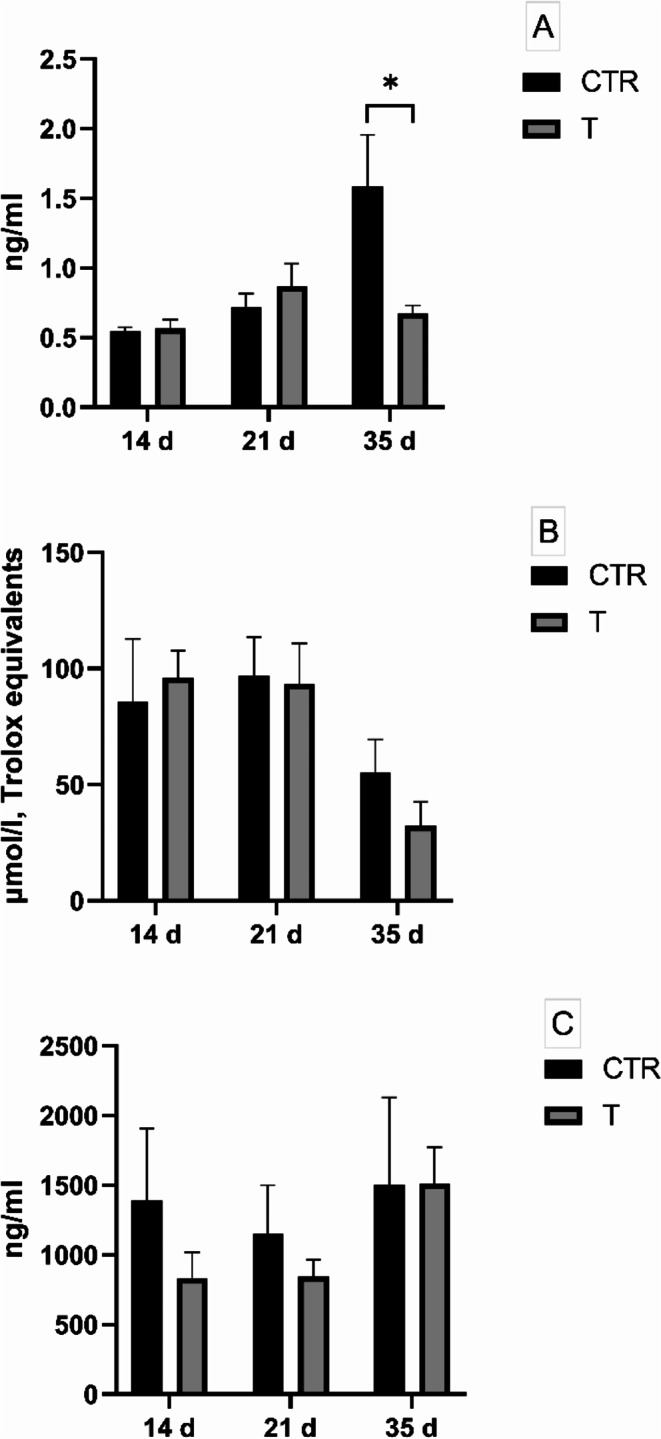
Salivary cortisol, TAC measured by FRAP and IgA (A, B and C respectively) registered at 14, 21, and 35 d on the trial in control (CTR) and treated (T) groups; *n* = 7 per group. Values are expressed as mean ± standard error mean (S.E.M). * = *P* < 0.05, ** = *P* < 0.01

At day 35d, a significative decrease in salivary cortisol was highlighted in the T group (0.672 ± 0.16 ng/ml vs. 1.589 ± 1.03 ng/ml, *P* < 0.05, Fig. 3A). No differences were detected in salivary IgA levels (Fig. 3B) and total antioxidant capacity between CTR and T group during the trial (Fig. 3C).

### Intestine histology and histometry

**Fig. 4:**
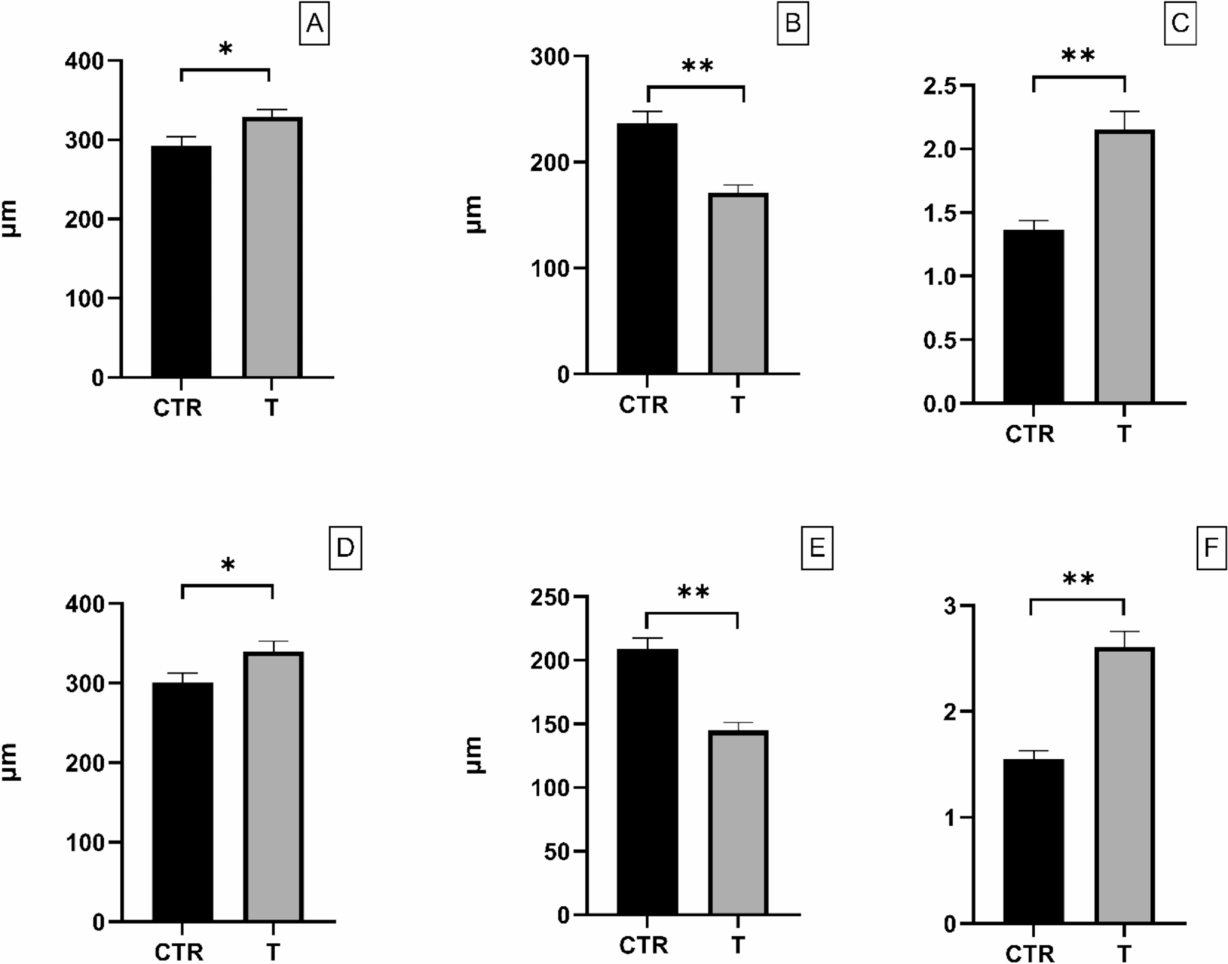
Villi height, crypt depth and V/C ratio registered in duodenum (A, B and C respectively) and Jejunum (D, E and, F respectively) at 35d in control (CTR) and treated (T) groups. Values are expressed as mean ± standard error mean (S.E.M). * = *P* < 0.05, ** =P < 0.01

Gut morphology of both control and treated animals was revealed to be structurally normal, with no sign of inflammation or epithelial detachment. Moreover, duodenal morphology was affected by the inclusion of the tested blend in the piglet’s diet. A higher villi height was registered in the T group when compared with CTR (320.27 ± 68.53 μm vs. 292.33 ± 90.35 μm; *P* < 0.05, Fig. 4A). On the other hand, deeper crypts were evident in CTR samples (171.38 ± 50.56 μm vs. 236.45 ± 90.38 μm; *P* < 0.01, Fig. 4B). Consequently, the V/C ratio highlighted higher values for T samples when comparing them to CTR (2.16 ± 1.04 vs. 1.37 ± 0.56; *P* < 0.01, Fig. 4C). Jejunum revealed comparable results when analyzing the morphometric characteristics. Indeed, samples of the T group showed increased villi height (339.94 ± 107.76 μm vs. 300.66 ± 101.74 μm; *P* < 0.05, Fig. 4D), decreased crypts depth (144.83 ± 53.40 μm vs. 208.82 ± 73.00 μm; *P* < 0.01, Fig. 4E) and a higher V/C ratio (2.61 ± 1.21 μm vs. 1.55 ± 0.65 μm; *P* < 0.01, Fig. 4F).

### Gut barrier assessment: E-Cadherin, Zonulin-1, and Occludin immunofluorescence staining

**Fig. 5:**
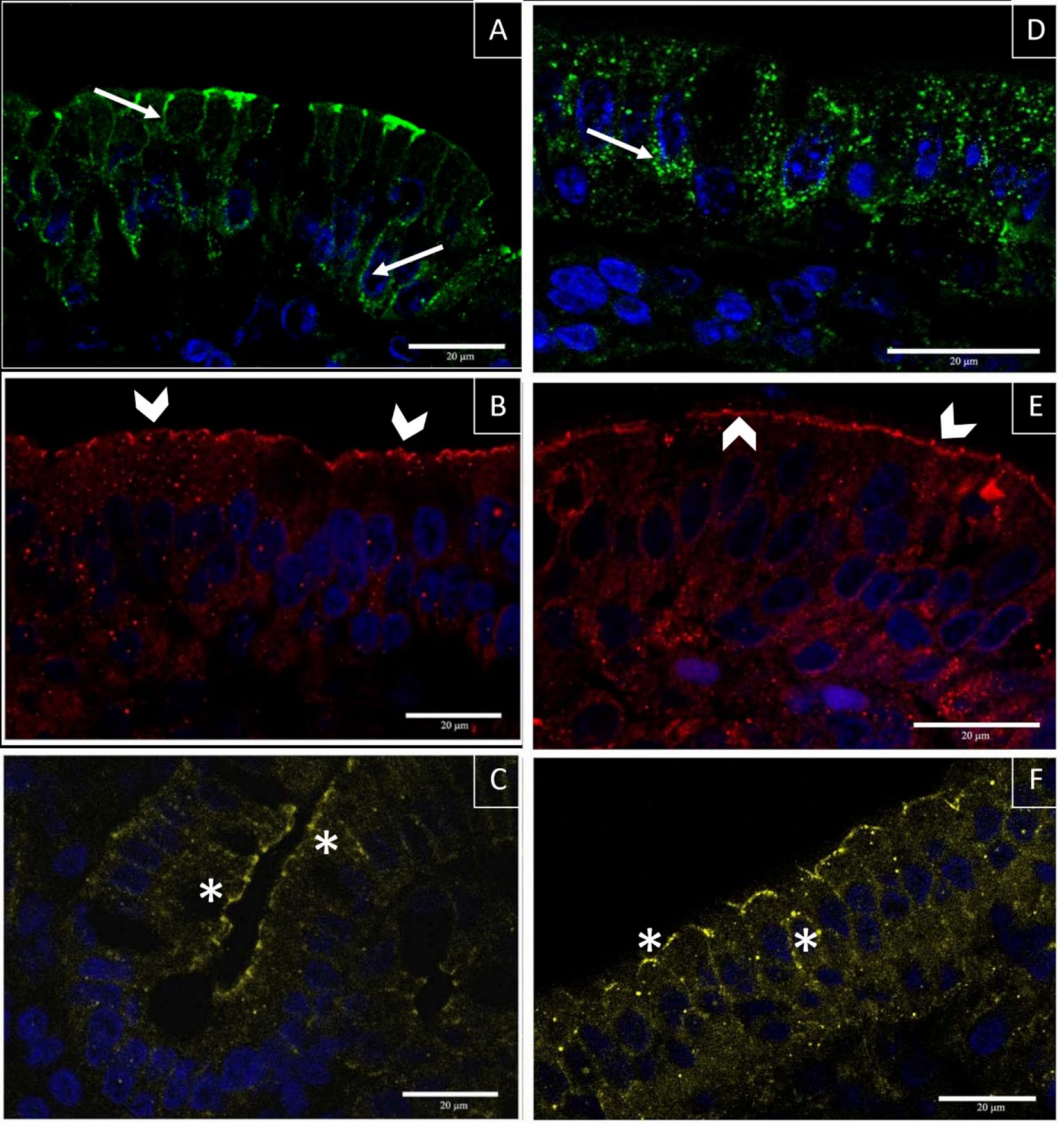
Representative images of immunofluorescence (IF) of E-Cadherin (A, D), Zonulin-1 (B, E), and Occludin (C, F) in treated animals in the duodenum (A, B, C) and in the jejunum (D, E, F). E-Cadherin IF in green, indicated by arrows; ZO-1 IF in red, indicated by bold arrows; Occludin IF in yellow, indicated by asterisks; nuclei, blue. Scale bar located in each image: 20 μm

Junction proteins were specifically assessed along the length of the villi in both duodenum and jejunum of treated animals. E-Cadherin was mostly found in cell-cell contact junctions in both intestinal tracts (Fig. 5A, duodenum and D, jejunum, green colour, arrows), throughout the length of the villi. Distinctly, ZO-1 was located only at the apical end of the enterocytes in both the duodenum and the jejunum (Fig. 5, B and E respectively, red color, bold arrows). Finally, Occludin was observed at apical and basolateral plasma membrane domains, either in the duodenum or the jejunum (Fig. 5C and F, respectively, yellow colour, asterisks).

**Fig. 6:**
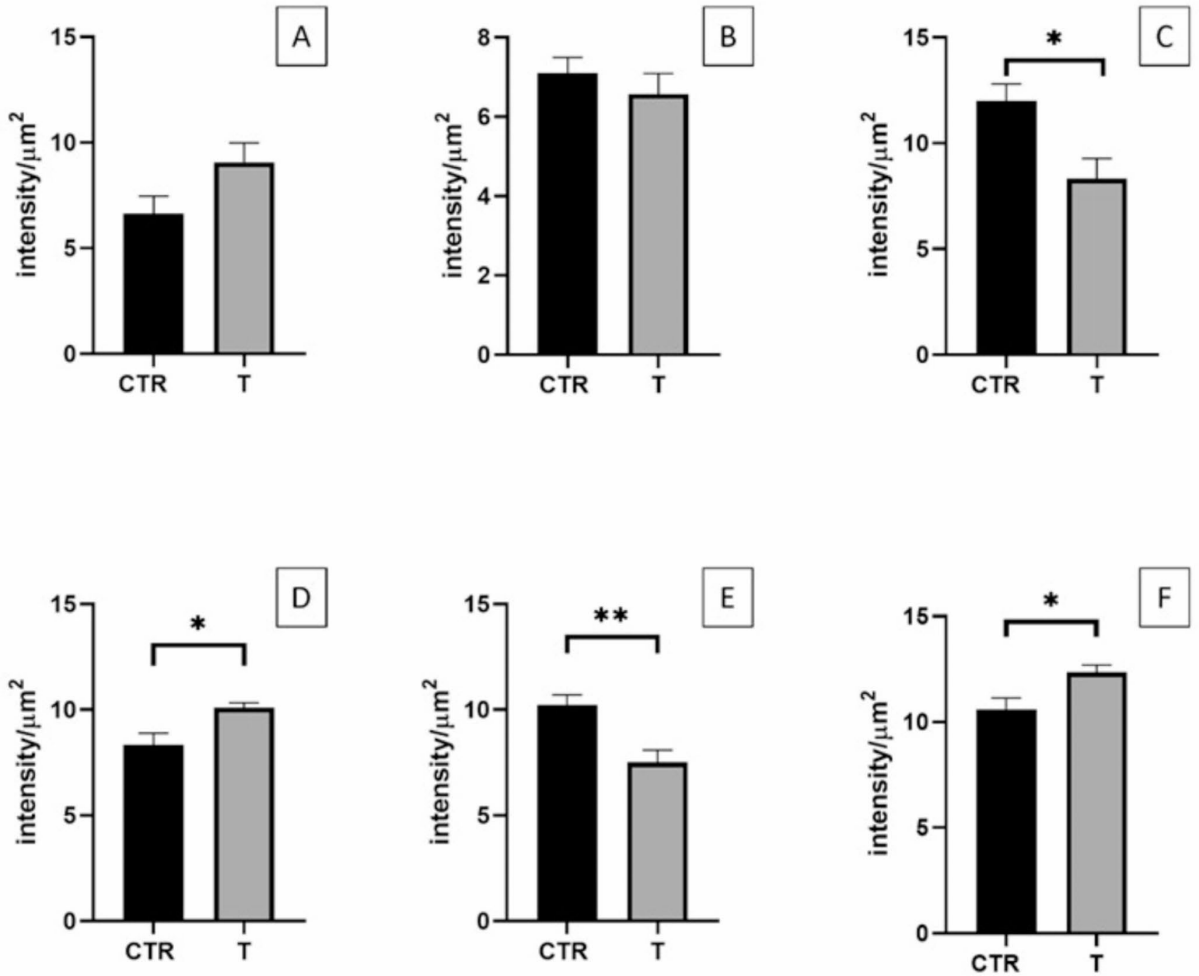
Quantitative representation of the expression of E-Cadherin in the duodenum (A) and in the jejunum (D), of Zonuline-1 in the duodenum (B) and in the jejunum (E), and of Occludin in the duodenum (C) and in the jejunum (F) of control (CTR) and treated (T) groups sampled at 35 d. Values are expressed in intensity per µm^2^. One-way ANOVA was performed. Results are expressed as mean ± standard error mean (S.E.M). * = *P* < 0.05, ** = *P* < 0.01

Junction protein expression was also quantified (Fig. 6). At the duodenum level, statistical differences were found only with Occludin, where the control animals showed a higher expression (Fig. 6C, *P* < 0.05). Regarding the jejunum, statistical differences were found for all three staining. Animals of the treated group showed higher expressions of E-Cadherin and Occludin (Fig. 6D and F respectively, *P* < 0.05 ), while with Zonulin-1, the result was the opposite (Fig. 6E, *P* < 0.01).

### Caecal microbiota

**Fig. 7:**
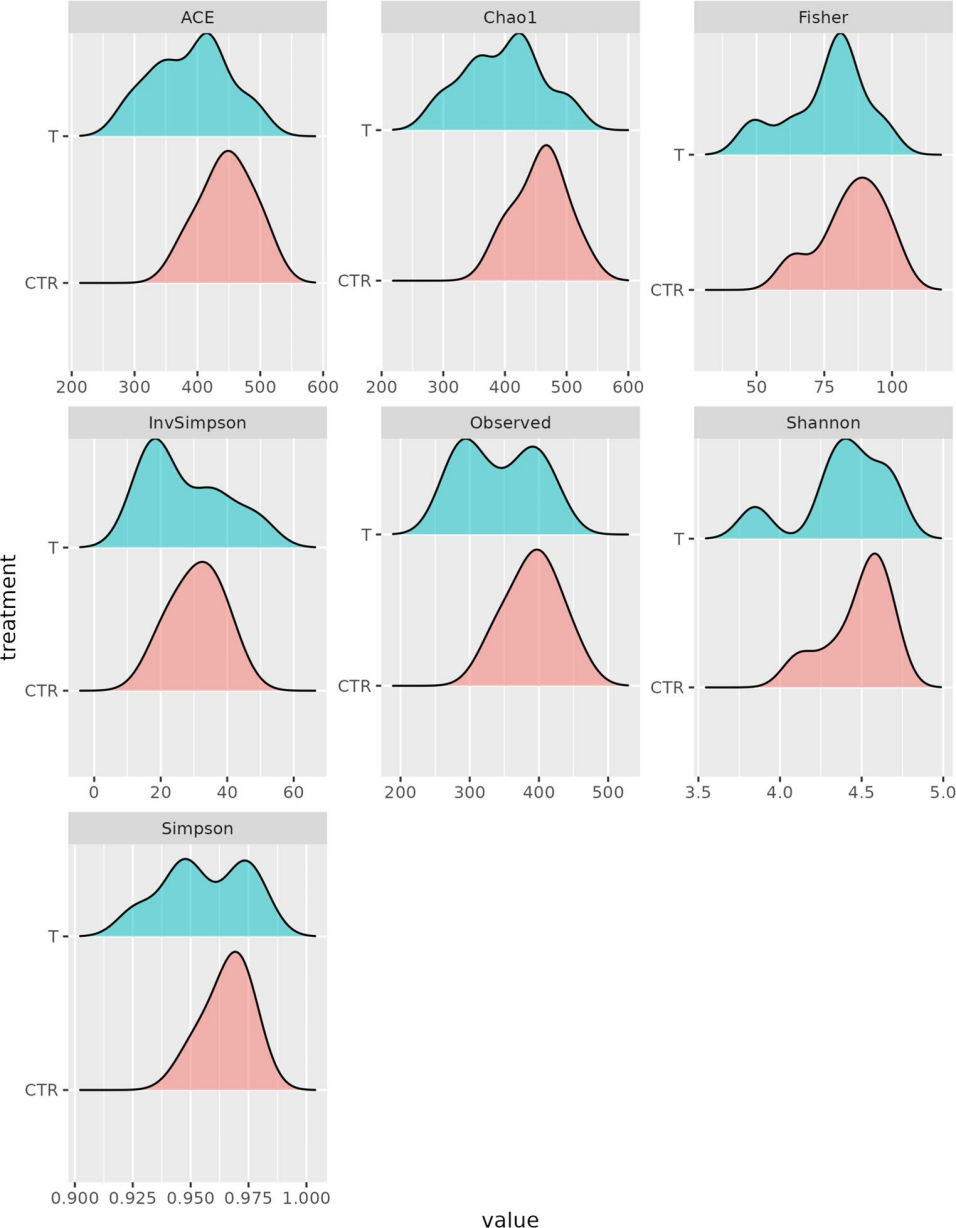
Density graph referred to alpha diversity evaluation performed on caecal samples of control (CTR) and treated (T) groups collected at the end of the trial (35 d)

**Fig. 8:**
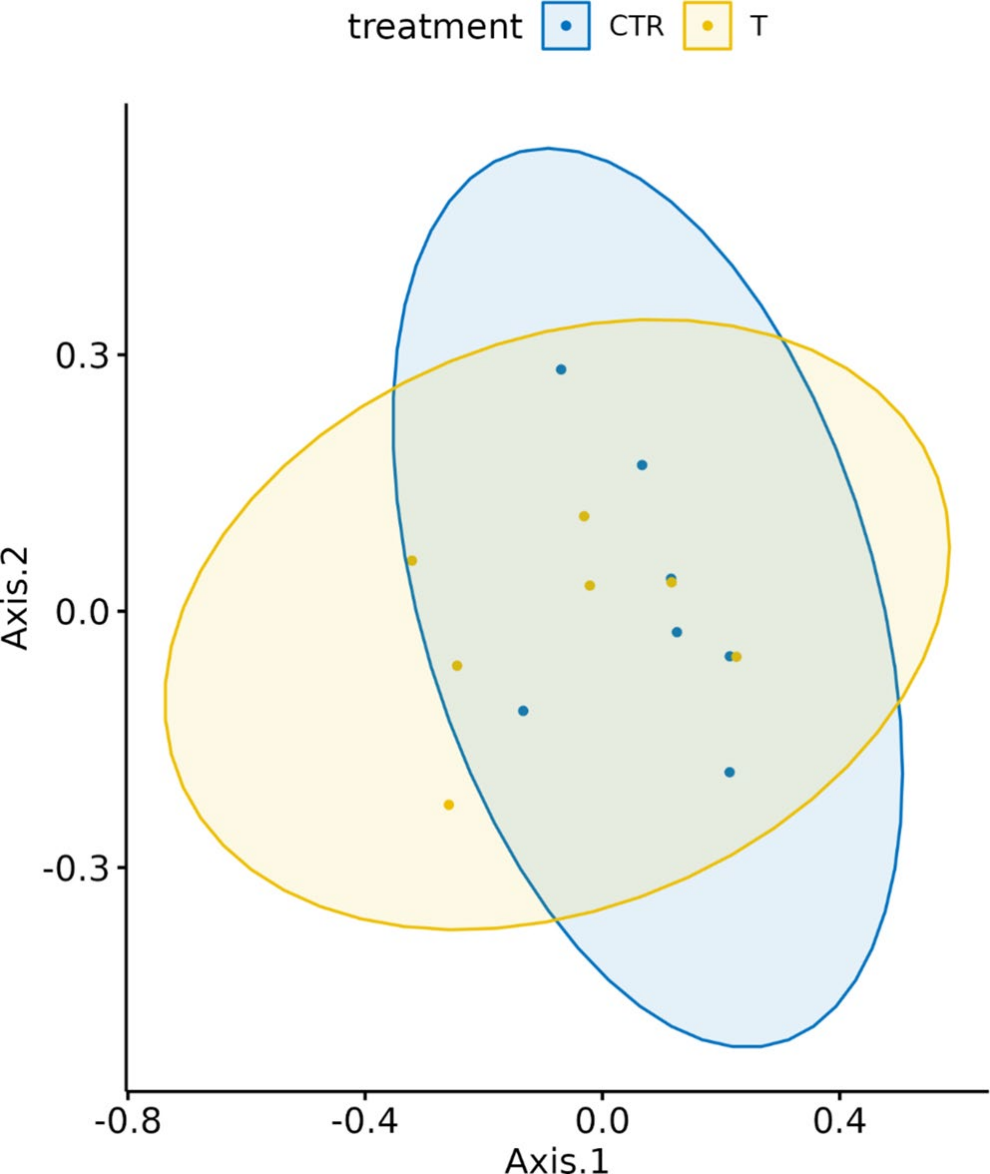
Ellipse graph referred to Bray-Curtis dissimilarities of control (CTR) and treated (T) groups evaluated on caecal samples collected at the end of the trial (35 d)

The gut microbial diversity was assessed within- (alpha diversity) and between- (beta diversity) samples (Fig. 7). All indexes for alpha were estimated from the complete OTU, filtered for OTUs with more than 10 total counts distributed in at least three samples and normalized for uneven sequencing depth by cumulative sum scaling (CSS). Within-sample microbial richness and diversity were estimated using the following indices: Chao1 and ACE (Abundance-based coverage Estimator) for richness and, on the other hand, Shannon, Simpson for evenness and Fisher’s alpha for diversity. Beta diversity was estimated based on Bray-Curtis distances (Fig. 8). None of the differences between treated and control samples for the diversity indices was significant (all *p*-values > 0.05). From PERMANOVA, the *p*-value for the treatment effect was 0.093. Therefore, both alpha and beta diversity of the piglets’ gut microbiota were not significantly affected by the treatment.

**Fig. 9:**
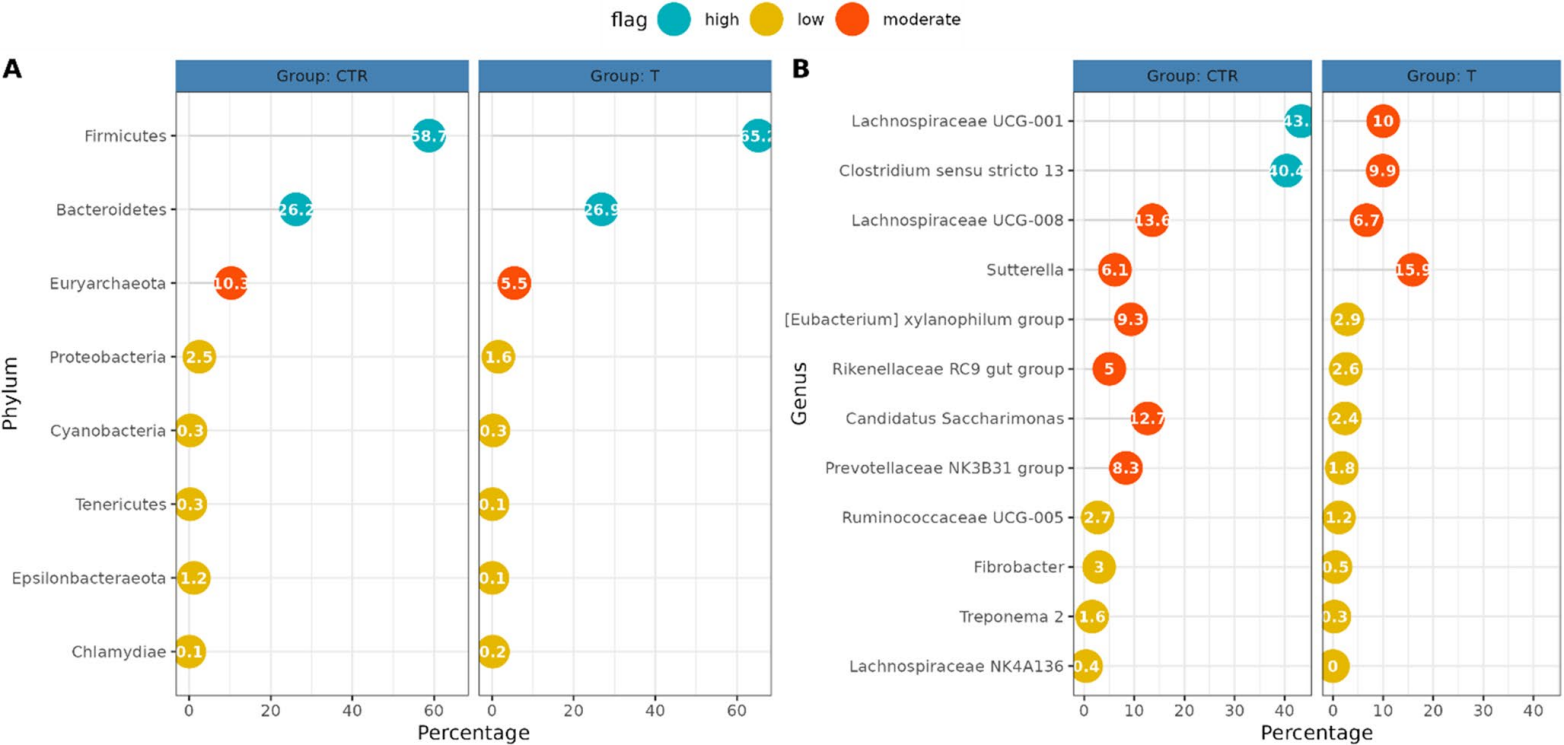
Percentage of average counts of phyla (A) and genera (B) detected in the two experimental groups. None of the differences between treatments for the reported phyla were significant (*P* > 0.05). On the other hand, 12 abundant genera were affected by dietary treatment (*P* < 0.05). CTR: control group; T: treated group

The 16 S rRNA gene sequencing results from all caecal samples were used to define the core microbiota of piglets. OTUs were taxonomically organized in phyla and genera. Sequencing the V3-V4 regions of the bacterial 16 S rRNA gene produced a total of 2,872,968 reads after filtering for quality. The resulting OTU table contained 729 OTUs that reduced to 409 OTUs after filtering for abundance and distribution. Phyla with relative abundance lower than 0.1% were not considered. A total of 12 abundant genera were significantly affected by the dietary treatment. In particular, the administration of the blend affected genera such as *Lachnospiraceae UCG-001*, *Lachnospiraceae UCG-008*, *Prevotellaceae NK3B31* group, *Clostridium sensu stricto 13* and *Ruminococcaceae UCG-005* (*P* < 0.05). Phyla and genera average counts are displayed in Fig. 9 (A and B respectively).

## Discussion

The blend considered in this study is composed of carvacrol, tannic acid (TA) from *Castanea sativa* mill, *Glycyrrhiza glabra* extract, diglycerides and triglycerides of medium-chain fatty acids (C:8, C:10, and C:12; MCFAs). Carvacrol is a monoterpene phenol originally found in different varieties of *Origanum*, *Thymus*, and *Satureja* plants (Suntres et al. 2015). Carvacrol supplementation in animal feed is a valid strategy to promote gut health due to its antioxidant, anti-inflammatory, and antimicrobial properties (Alagawany et al. 2015). MCFAs are molecules characterized by the presence of 6 to 12 carbon atoms (Ferronato and Prandini 2020). Lauric, capric, and caprylic acid are naturally present in palm and coconut oil (Jadhav and Annapure 2023). As reported by Rebucci et al. (2021) to avoid the complete absorption of their free forms at the gastric level, MCFAS are administered as saponified or glycerides to promote gut health in the enteric tract without being directly dissociated in the gastric tract. Tannic acid (TA) is a naturally occurring and hydrolysable polyphenolic compound, characterized by the presence of heterogeneous phenol groups available to different molecular interactions (Watrelot et al. 2020). As in the case of MCFAs, TA bactericidal activity seems to be more effective towards gram-positive bacteria (Baldwin and Booth 2022). Blended feed additives recently gained an increasing interest in the feeding of post-weaning piglets. Moreover, the study of blends in livestock nutrition could be crucial to optimize the effectiveness of these molecules under varying conditions (Abdelli et al. 2021). Furthermore, blends characterized by different compositions may display positive effects on the intestinal environment of weanling piglets, contributing to preventing the reduction of antibiotics and representing an alternative to pharmacological dosages of ZnO (Luise et al. 2023). An in vitro characterization of the depicted blend anticipated the in vivo phase of the present study. Briefly, an overlapping trend was depicted when looking at TPC and antioxidant capacity over the digestion process (oral, gastric, and intestinal). Interestingly, the trend highlighted a peak after gastric digestion in TPC, FRAP, and ABTS assays which was followed by a marked decrease after the intestinal phase. The marked increase of TPC after the gastric phase might be due to the efficacy of acidic pH in breaking the polysaccharide bonds and the subsequently release of phenol compounds which are associated with antioxidant capacity as depicted by Lanzoni et al. (2023). As reported by the author, the significant decrease observed after the intestinal phase can be related to the instability of phenols in the intestinal environment (basic pH and/or pancreatic enzymes effect). Nevertheless, the antioxidant activity registered at the end of the digestion together with the high MCFAs content in the tested blend (23%) could highlight the positive effects further evidenced during the in vivo trial.

The first effect of the dietary blend inclusion was registered on animals performance. Shao et al. (2023) found an increased ADG and ADFI during the first 4 weeks after weaning in piglets fed a blend of essential oils containing 2.34% of carvacrol. Moreover, Cui et al. (2020) registered better performances when considering different glycerides forms and combinations of lauric, capric, and caprylic MCFAs. Furthermore, despite the potential detrimental effect of tannins on performances due to limited protein digestibility, contained TA supplementation (1.13, 2.25, and 4.25 g/kg) enhanced feed efficiency during the weaning transition (Biagia et al. 2010). In our study, the blend of carvacrol, TA, and MCFAs improved the ADG and BW of weaned piglets, confirming the beneficial potential of these molecules reported in the literature. Although the effect of MCFAs and carvacrol has not been reported to constantly ameliorate the fecal consistency of post-weaning piglets alone or in combination with natural extracts (López-Colom et al. 2020), it has been previously reported how TA and commercial tannins in general are useful tools to increase fecal score (Girard et al. 2018). Given that, the fecal score registered suggested a positive interaction of the different components in increasing the fecal consistency in critical stages of the post-weaning phase.

Saliva has been depicted to have great potential in displaying stress and disease characterization through the detection of stress, innate and adaptive immune response, and oxidative stress biomarkers in pigs (Sánchez et al. 2022). Moreover, salivary secretory immunoglobulin A is considered a natural defence for the host (Pedersen et al. 2019). In our study, no significant differences were found between CTR and T group in salivary IgAs. As reported by Muneta et al. (2010), Salivary IgAs could be influenced both by the circadian rhythm and feeding. In addition, Svobodová et al. (2014) indicated that the role of salivary IgAs was not confirmed when considering the short or long-term stress stimuli associated to weaning. Switching attention to salivary total antioxidant capacity (TAC), Saco et al. (2023) recently reported how this non-invasive biomarker could be strongly influenced by the production phase in addition to the circadian rhythm. According to Sánchez et al. (2022), there is a strong relationship between adaptive immune and oxidative stress salivary biomarkers. Perhaps, this could explain the absence of variations in the salivary TAC values registered in our trial when comparing the experimental groups. Given that, considering the lack of consistent pieces of evidence regarding the application of salivary IgAs and TAC in weanling piglets’ nutritional studies, it can be stated that the variability of these two salivary biomarkers needs further investigation. Salivary cortisol has been confirmed as a reliable biomarker capable of describing the physiological conditions of pigs during critical phases of the production cycle (Bahnsen et al. 2021). Zhang et al. (2023) found that 50 mg/kg of a mixture of carvacrol, thymol, and cinnamaldehyde in a 1:1:1 proportion complex did not reduce cortisol levels in weanling piglets’ blood. Furthermore, the lack of results in conditioning salivary cortisol levels through natural extracts and essential oils administration was previously underlined when considering different concentrations of garlic powder (0.4% and 1.2%) and oregano essential oil (0.4% and 2.0%), even though authors detected lower concentrations of serum cortisol of the piglets involved in the same study (Rivera-Gomis et al. 2020). On the contrary, in our study we detected a marked decrease of cortisol levels in saliva samples 35 d after weaning, suggesting a stimulation of the hypothalamic-pituitary-adrenal (HPA) axis, responsible for the glucocorticoid hormone production. Briefly, as depicted by Moeser et al. (2007) weaning stress is associated with the enhancement of the corticotropin-releasing factor (CRF) expression, which is linked to gut barrier dysfunctions. Due to this, it is reasonable to relate the physiological changes and stress that occur during the weaning transition to variations in cortisol levels (Wu et al. 2023) which served as indirect biomarker of gut barrier integrity. Therefore, our results suggested a key role of blended carvacrol, TA, and MCFAs blend in lowering cortisol levels which served as indirect biomarker of gut health, as depicted also by further discussed results.

The ability of the tested blend in conditioning gut morphology was assessed through duodenal and jejunal histology and histometry. Mo et al. (2023) recently showed the capability of a 5% carvacrol, 2% thymol, and 3% cinnamaldehyde blend to enhance the villus height and crypt depth ratio in the jejunum of weaned piglets. Furthermore, a blend of 1 g of both capric and caprylic acids modified the small intestine mucosal structure of weanling piglets decreasing crypt depth as depicted by Hanczakowska et al. (2011). On the other hand, Ferrara et al. (2016) underlined a lack of effects when considering the 0.15% of both capric and caprylic acids blended with short-chain fatty acids in the jejunal morphometry. Moreover, when blending 10.1% calcium formate 25.1% of citric acid, and an essential oils mixture (4.5% thymol, 4.8% carvacrol and 4.3% cinnamaldehyde) it is possible to increase villi height and influence V/C ratio as reported by Liu et al. (2022). Our results are in line with this picture, testifying an increased villus height and V/C ratio both in duodenum and jejunum, but also remarking a significantly enhanced crypt depth for the CTR group. Chwen et al. (2013) reported that deeper crypt depth is associated with a more intense turnover of the enterocytes, which could further result in higher villi. However, not always higher length villi are associated with an increased absorptive capacity. According to Pluske et al. (1997), the intestine may show lower absorptive capacity even with high villi length, when the enterocytes are not mature. It does not seem to be our case, considering the other positive results obtained in this study, such as body weight, fecal score, and saliva cortisol. Moreover, there are indications that, in germ-free animals, the increased villi height does not necessarily correspond to an increase in the absorptive capacity (Williams et al. 2015), but this is neither the case of our study. Thus, it is reasonable to speculate that the tested blend positively influenced the morphometric characteristics of the proximal intestine. Our results may be representative of a situation in which the treatment allowed a more rapid recovery of small intestine tissue conditions after weaning than in the control group.

TJ are complex structures comprising over 50 proteins and include a series of transmembrane proteins, such as Occludin and Zonulin. Adherens junctions, such as E-Cadherin, are located beneath the TJ and are involved in cell-cell adhesion and intracellular signaling, and all together these protein junctions regulate paracellular permeability (Krug and Fromm 2020). E-Cadherin is an adherents junction molecule, which plays a key regulatory role in barrier integrity through its temporal and spatial coordination of the tight junction (Itoh et al. 1997; Umeda et al. 2006). Additionally, Zonulin-1 is the major TJ protein and in normal intestines, it is expressed exclusively at the apical TJ (Fasano 2012). Furthermore, occludin is an integral membrane protein of epithelial tight junctions (Anderson and Van Itallie 2009; Wu et al. 2020).

Positive results were obtained by Zhao et al. (2023) considering duodenal Occludin and ZO-1 mRNA expression after the administration of carvacrol–cinnamaldehyde–thymol blend. On the contrary, Wei et al. (2017) found no effects when considering the supplementation of a blend of carvacrol and thymol essential oils at 100 mg/kg (1:1) on ZO-1 and Occludin expression in jejunal mucosa. In addition, a mixture of natural extracts was useful in enhancing the expression of E-Cadherin in the distal enteric tract of weaned piglets (Su et al. 2018). Briefly, Grilli et al. (2016) showed that sodium butyrate reduced the expression of claudin-1 in the duodenum and jejunum, while Occludin was regulated in the duodenum but not in the jejunum tract. Focusing on protein abundance, Zou et al. (2016) obtained positive results administering essential oils to weaned piglets when looking at the Occludin and ZO-1 expression in the jejunum tract. Moreover, Cui et al. (2020) registered an increased jejunal Occludin expression by administering 2 g/kg of glycerol monolaurate in weanling piglets. On the contrary, the author reported a lack of effects on ZO-1 and Occludin after two weeks of administration of tricaprylin and tricaprin mixture (1 g/kg each). Furthermore, a phytobiotic mixture derived from oregano extract composed of carvacrol and thymol showed no effects on ZO-1 and a significant decrease in Occludin levels in jejunum 21 d after weaning (Duarte and Kim 2022). The localization of the three molecules performed in our study is consistent with what is described in the literature, in particular, regarding E-Cadherin in cell-cell contact junctions throughout the length of the villi (Hwang et al. 2012; Xu et al. 2008; Zahn et al. 2008), ZO-1 at the apical end of the enterocytes (Dong et al. 2021; Fasano 2011; Kimura et al. 1997; Nouri et al. 2014; Aidos et al. 2023), and Occludin at apical and basolateral plasma membrane domains (Dong et al. 2021; Kimura et al. 1997). From the quantification of the protein expression, it seems that the jejunum is more susceptible to diet interventions, as differences in the expression of all TJs studied, E-Cadherin, Zonulin-1, and Occludin showed statistically significant differences in this tract. This is probably because the jejunum is the major site for nutrient absorption, while the duodenum is mostly dedicated to digestion (Campbell et al. 2019). However, in the duodenum, there was a difference in the expression of Occludin, which was opposite to the one observed at the jejunum level. Considering the duodenum and its digestive functions, it is reasonable to think that this result was conditioned by the presence of pancreatic enzymes and bile. Indeed, it is recognized how variations in pancreatic enzymes and bile salts can influence the bio accessibility of phytochemicals (Wojtunik-Kulesza et al. 2020). Considering the expression results of jejunal E-Cadherin and Occludin, it can be noticed that the treated animals showed a lower intestine permeability when compared with the control group animals, outlining a protective effect of the treatment on the intestinal barrier. However, the expression of ZO-1 showed an opposite trend: treated animals showed lower ZO-1 expression. The explanation for this results may be found in the role of Zonulin as an endogenous mediator in the physiological regulation of intercellular tight junctions in the small intestine (Fasano 2011, 2020; Fasano et al. 2000; Hałasa et al. 2017). Indeed, ZO-1 can reversibly modulate the permeability of the intestine (Fasano et al. 2020). For instance, an increase in the expression of zonulin with a subsequent increase in permeability, was observed in human intestinal diseases, like irritable bowel syndrome, non-celiac gluten sensitivity, environmental enteropathy, and necrotizing enterocolitis (Sturgeon et al. 2016). Therefore, the higher expression of ZO-1 in the control group observed in this study may indicate an alteration in intestinal permeability in these animals.

Since abrupt weaning-related changes in the gut microbial core can negatively affect homeostasis, epithelia turnover and gut barrier functions with negative reflexes on general gut health (Ren et al. 2022; Blachier et al. 2017) caecal microbiota was evaluated in the presented study. Briefly, at genus level, modulation of *Ruminococcaceae*, *Rikenellaceae* and *Prevotellaceae* genera was registered. *Lachnospiraceae* has been previously linked to short chain fatty acids production, with possible positive reflexes on gut and host health (Jiangh et al. 2021). On the other hand, its role in piglets gut environment is still controversial and not clear (Wang et al. 2022). In addition, *Rikenellaceae* group displayed a potential role of intestinal health biomarker and was positively correlated with higher feed convertion rate (Quan et al. 2018). Briefly, *Prevotellaceae* NK3B31 and *Ruminococcaceae* UCG-005 have been link to the production of acetate and propionic acid derived from resistant starch degradation, as in the case of *Fibrobacter* which was linked to cellulose digestion in diverse hindgut fermenters (Wang et al. 2019; Gaukroger et al. 2020). Controversary findings were highlighted regarding the role of *Candidatus Saccharimonas*, which was found less abundant both in high and low feed efficiency pigs and high abundant in caecal samples of obese pigs in a metabolome-microbiota relation trial (Liu et al. 2023). Moreover, even without evidence about *Eubacterium xylanophilum* group presence and development in piglet gut during weaning, rumen isolates of *Eubacterium xylanophilum* were reported to degrade xylan and produce short chain fatty acids. Treponema 2 sequences have been linked to higher feed efficiency, lignin degradation and higher presence of *Methanobrevibacter* in large intestine of pigs (Gardiner et al. 2020). Regarding *Sutterella*, in humans this genus has been previously correlated to inflammatory bowel diseases conditions and IgA degradation (Shapiro et al. 2021). On the contrary, more recently *Sutterella* has been positively related to feed intake and lipids digestion in commercial hybrid pigs (Luo et al. 2022). *Sutterella* along with *Proteobacteria* was found to be particularly abundant in diarrheic Tibetan early weaned piglet by Kong et al. (2022). Furthermore, an increasing number of studies are relating *Clostridium sensu stricto* 13 to inflammatory enteric diseases and diarrhea (Wang et al. 2023; Chen et al. 2022). In our case, despite a reduced presence of *Ruminococcaceae*, *Lachnospiraceae* and *Prevotellaceae*, the treated group registered a lower abundance of *Clostridium sensu stricto* 13. Considering what was previously assessed, it is more probable that the direct effect of the blended compounds on duodenal and jejunal histometry and gut barrier integrity reflected a reduction of *Clostridium sensu stricto* 13 at 35 d rather than directly act on its abundance. Therefore, it is reasonable to link the reduction of *Clostridium sensu stricto* 13 to a better status of the intestinal barrier.

## Conclusions

The administration of a blend composed of Carvacrol, tannic acid, and MCFAs improved the gut health of weaned piglets ameliorating fecal consistency, physiological stress status, gut tissue morphometry and permeability, and reflecting changes in *Clostridium sensu stricto* 13 abundance. Nevertheless, differences in compositional terms of the compared blends must be considered. In conclusion, further research is needed to elucidate the synergistic effect and different inclusion levels of these substances when applied in commercial farm conditions.

## Data Availability

Upon reasonable request, data are available from the corresponding author: luca.marchetti1@unimi.it.
